# A novel cell-free method to culture *Schistosoma mansoni* from cercariae to juvenile worm stages for *in vitro* drug testing

**DOI:** 10.1371/journal.pntd.0006590

**Published:** 2019-01-28

**Authors:** Sören Frahm, Anisuzzaman Anisuzzaman, Ulrich Fabien Prodjinotho, Nermina Vejzagić, Admar Verschoor, Clarissa Prazeres da Costa

**Affiliations:** 1 Institute for Medical Microbiology, Immunology and Hygiene, Technische Universität München, Munich, Germany; 2 Department of Parasitology, Bangladesh Agricultural University, Mymensingh, Bangladesh; 3 Institute for Systemic Inflammation Research, Universität zu Lübeck, Lübeck, Germany; McGill University, CANADA

## Abstract

**Background:**

The arsenal in anthelminthic treatment against schistosomiasis is limited and relies almost exclusively on a single drug, praziquantel (PZQ). Thus, resistance to PZQ could constitute a major threat. Even though PZQ is potent in killing adult worms, its activity against earlier stages is limited. Current *in vitro* drug screening strategies depend on newly transformed schistosomula (NTS) for initial hit identification, thereby limiting sensitivity to new compounds predominantly active in later developmental stages. Therefore, the aim of this study was to establish a highly standardized, straightforward and reliable culture method to generate and maintain advanced larval stages *in vitro*. We present here how this method can be a valuable tool to test drug efficacy at each intermediate larval stage, reducing the reliance on animal use (3Rs).

**Methodology/Principal findings:**

Cercariae were mechanically transformed into skin-stage (SkS) schistosomula and successfully cultured for up to four weeks with no loss in viability in a commercially available medium. Under these serum- and cell-free conditions, development halted at the lung-stage (LuS). However, the addition of human serum (HSe) propelled further development into liver stage (LiS) worms within eight weeks. Skin and lung stages, as well as LiS, were submitted to 96-well drug screening assays using known anti-schistosomal compounds such as PZQ, oxamniquine (OXM), mefloquine (MFQ) and artemether (ART). Our findings showed stage-dependent differences in larval susceptibility to these compounds.

**Conclusion:**

With this robust and highly standardized *in vitro* assay, important developmental stages of *S*. *mansoni* up to LiS worms can be generated and maintained over prolonged periods of time. The phenotype of LiS worms, when exposed to reference drugs, was comparable to most previously published works for *ex vivo* harvested adult worms. Therefore, this *in vitro* assay can help reduce reliance on animal experiments in search for new anti-schistosomal drugs.

## Author summary

Schistosomiasis remains a major health threat, predominantly in developing countries. Even though there has been some progress in search of new drugs, praziquantel remains the only available drug. Probably the most important advance in the search for new drugs was *in vitro* transformation of cercariae and their subsequent culture. However, hit identification in compound screenings is exclusively tested in skin stage parasites and is only confirmed for more mature worms in a subsequent step. This is in part due to the lack of an easy culture system for advance-stage parasites. We present here a reliable and highly standardized way to generate LiS worms *in vitro* in a cell-free culture system. The inclusion of *in vitro* drug tests on advanced-stage parasites in initial hit identification will help to identify compounds that might otherwise be overlooked. Furthermore, the ability to continuously observe the parasite’s development *in vitro* will provide an important platform for a better understanding of its maturation in the human host. Taken together, this opens up new avenues to investigate the influence of specific cell types or host proteins on the development of *Schistosoma mansoni* and provides an additional tool to reduce animal use in future drug discovery efforts (3Rs).

## Introduction

Schistosomiasis, a chronic and debilitating helminthic disease, is one of the most important neglected tropical diseases (NTD). The WHO estimates that more than 206 million people are currently infected and in need of chemotherapy world-wide [1]. Moreover, over 200,000 people die each year due to the sequelae of the disease [2, 3]. Among the parasitic diseases, schistosomiasis is often considered only second in importance to malaria [2] and thus a major public health menace. Therefore, the WHO aims to eliminate schistosomiasis as a public health problem globally by 2025 [4, 5]. Implementation of safe water, sanitation and hygiene (WASH) strategies [6], intensified case management, veterinary public health, vector control and mass drug administrations (MDAs) are all crucial in reducing the disease burden [5]. Of all these approaches, MDA dominates national control programs thanks to the excellent safety and efficacy profile of praziquantel (PZQ), the only currently available drug [7, 8], as well as its low cost per treated individual [9]. However, the reliance on PZQ also raises concerns about emerging resistance should the drug pressure increase. Resistance against PZQ has already been observed in experimental models [10] while first instances of decreased drug efficacy have been observed in the field [7, 11, 12]. To be prepared for the emergence of resistant strains of schistosomes and to support the elimination of schistosomiasis, new drugs and complementary strategies, such as vaccines, are of imminent importance. To identify new drugs and new vaccine targets, *in vitro* assays of larval and adult worm stages are paramount for high throughput testing and simultaneously for reducing reliance on *in vivo* or *ex vivo* experiments in accordance with the 3Rs (replacement, reduction and refinement) of animal testing [13].

The current cultivation protocols for *in vitro* generated larval stages such as the schistosomula rely on the supplementation of fetal calf serum (FCS) for short-term culture [14, 15] and on the supplementation with FCS, human serum (HSe), erythrocytes and peripheral blood mononuclear cells (PBMCs) for long-term culture and *in vitro* juvenile worm development [16]. Such non-standardized culture conditions are prone to variability of serum batches, rely on the continuous supply of fresh human blood, and make it difficult to isolate pure schistosomula-derived soluble antigens during *in vitro* culture or to investigate the role of specific serum proteins in the development of the parasite. In addition to these hindrances and to avoid inter-assay or inter-laboratory fluctuations, well-defined and standardized culture conditions independent of serum and cell supplementations are needed. Up to now, long-term culture has been reported to rely on costly, non-commercially available and highly complex culture media such as Basch medium 169 which are difficult to implement in routine use and prone to batch variability [17]. Thus, simplifying culture conditions as well as the generation and handling of advanced-stage parasites opens new possibilities in the search for new drugs and facilitates the upscaling of existing drug screening strategies.

To establish a robust *in vitro* assay, it is important to continuously imitate the parasite’s *in vivo* development. This development within the final host is quite complex and occurs over a period of six to seven weeks. After penetration of the skin, cercariae transform to schistosomula which remain in the skin for about three days before migrating through the host’s vasculature. These skin stage schistosomules (SkS) traverse the capillaries of the lung where the majority of the parasites can be found on day 7 after infection [18]. To facilitate their migration, these SkS become longer, more slender and active and are hence called lung stage schistosomula (LuS). These continue their journey to the portal and mesenteric veins following the bloodstream [19, 20] where they continue to undergo morphological changes. The bifurcated gut is fused, the parasites initiate feeding, and continue to grow. This early liver stage (early LiS) is followed by the late LiS characterized by a drastic increase in length and prominent oral and ventral suckers. These juvenile worms (late LiS) then start to pair up and become fertile upon which the oviposition starts approximately 35 days after infection [21].

Treatment with PZQ, although initially with good efficacy, does not diminish the high reinfection rates encountered in the field [22]. This is partly due to the inability of PZQ to efficiently target early larval stages of the parasite [23, 24]. In the current search for new anti-schistosomal drugs, a two-step strategy is used. Firstly, SkS are tested immediately after their transformation from cercariae and then, once an active compound has been identified, it is tested mainly on *ex vivo* cultured adult worms that have been isolated from infected hamsters or mice [14, 25–27]. Other larval stages like the LuS, early and late LiS are omitted as potential drug targets. Therefore, future compounds with an activity predominantly directed against juvenile and adult stages might be overlooked. Thus, a highly standardized and robust way to generate advanced larval stages of *Schistosoma mansoni* would provide an opportunity to incorporate initial advanced-stage schistosomula testing into the current drug screening strategies to meet the desired target candidate profiles (TCP).

We disclose here a new serum- and cell-free cultivation method of newly transformed schistosomula (NTS) up to the LuS (7 days p.t.) and a cell-free cultivation method up to late LiS worms (starting to show 28 days after transformation) of *S*. *mansoni*. Our culture system allows the detection of stage-dependent differences in the activity of drugs with known anti-schistosomal properties, such as PZQ, Oxamniquine (OXM), the standard drug to treat schistosomiasis caused by *S*. *mansoni* prior to the advent of PZQ, and two antimalarial drugs that have recently been described to have anti-schistosomal properties, Mefloquine (MFQ) and Artemether (ART) [16, 28]. Therefore, this highly standardized, straightforward and reliable culture method is a basis for integrating drug screenings of advanced larval stages into initial hit identification in the search of novel schistosomicidal drugs.

## Materials and methods

### NTS generation

Cercariae of an in-house Brazilian or imported NMRI strain of *S*. *mansoni* were harvested from infected *Biomphalaria glabrata* snails and used for mechanical transformation into NTS as described before [29]. *Biomphalaria glabrata* snails infected with the NMRI strain were provided by the NIAID Schistosomiasis Resource Center of the Biomedical Research Institute (Rockville, MD) through NIH-NIAID Contract HHSN272201700014I for distribution through BEI Resources and used for experiments as indicated due to temporary limited availability of the Brazilian strain. Briefly, cercariae were incubated 30 min on ice then centrifuged at 1932 x g for 3 min at 4°C. The pellet was resuspended in Hank’s balanced salt solution (HBSS) (Cat. No. H6648, Sigma-Aldrich, Germany) supplemented with 200 U/ml Penicillin and 200 μg/ml Streptomycin (Cat. No. P4333, Sigma-Aldrich) and transformed by mechanical stress applied by pipetting and vortexing, which was confirmed by microscopy (10x magnification). Separation of tails and cercarial bodies was accomplished by repeated sedimentation in ice-cold HBSS. The NTS were then transferred to the various culture media and kept overnight to complete transformation.

### NTS culture

NTS (~100 NTS in 150 μl) were maintained in HybridoMed Diff 1000 (HM) (Cat. No. F 8055/1, Biochrom GmbH, Germany), Medium 199 (M199) (Cat. No. M4530), Dulbecco’s Modified Eagle Medium (DMEM) (Cat. No D5796) or RPMI 1640 (Cat. No. R8758, Sigma-Aldrich, Germany) supplemented with 200 U/ml penicillin, and 200 μg/ml streptomycin (Sigma-Aldrich) in a 96-well flat bottom tissue culture plate (Cat. No. 353075, Corning Incorporated, USA) and incubated at 37°C in 5% CO_2_ and humidified air for up to 4 weeks. For each condition, experiments were performed in triplicates. The medium was replaced every 7 days. Scoring was performed on day 1, 3 and 7 post-transformation (p.t.) and then again weekly until week 4 p.t.

### Scoring criteria for the viability score

The viability of NTS was scored using an Axiovert10 microscope (Zeiss, Germany). The scoring system was adapted from the Swiss TPH [29] and the WHO-TDR [27]. For the scoring, three main criteria were assessed: motility, morphology and granularity. The score was applied ranging from 0 (dead parasites, no movement, heavy granulation, blurred outline, rough outer tegument and blebs) to 1 (very reduced motility, rough outer tegument with some blebs) to 2 (reduced motility or increased uncoordinated activity, slight granularity, intact tegument with slight deformations), and finally 3 (regular smooth contractions, no blebs and a smooth outer surface, no granulation with clear view of internal structures which are visible under bright field microscope). Single NTS die during the mechanical transformation and are, thus, present from the beginning. The amount of dead parasites is taken into consideration when applying the viability score, thereby lowering the overall score. The score represents an overall impression of the visual appearance of all NTS in a well. In order to capture any subtle changes in the appearance of all schistosomula per well, viability scores 0–3 were further subdivided into 0.25 steps (e.g. 0, 0.25, 0.50, 0.75). For determination of larval development stage, morphological characteristics were used and based on previously published works [17, 21]. The skin stage was characterized by resembling the cercarial head in shape and undirected regular movements, the lung stage by an increase in length and decrease in diameter. The early LiS was characterized by growth of the parasite initially in diameter and then further in length and the clear visibility of the gut. The late LiS showed clearly identifiable oral and ventral suckers and a further increase in length, especially of the body past the ventral sucker.

### Growth promotion with human serum

Blood sampling of HSe was prepared from blood of consenting healthy volunteers with no previous history of schistosomiasis upon written consent. Fresh blood was left at room temperature for 30 min to clot, then centrifuged at 1845 x g for 20 min and serum was collected and pooled from 6 individuals and stored at -20°C until further use. NTS were incubated (100 in 150 μl) in Basch-Medium 169 [30] kindly provided by Prof. C. Grevelding and Dr. T. Quack (Universität Giessen, Germany), DMEM and HM supplemented with 200 U/ml Penicillin and 200 μg/ml Streptomycin and with HSe in different concentrations (1–50%) or 20% heat-inactivated FCS (Cat. No. F7524, Sigma-Aldrich, Germany) at 37°C for 8 weeks in a 96-well plate. Medium without HSe supplementation served as controls. The medium was changed weekly, and viability was scored on day 1, 3 and 7 p.t. and then again, every 7 days. Developmental stages were determined by bright field microscopy using an inverted Axiovert 10 microscope (Zeiss). For each condition experiments were performed in triplicates.

### *In vitro* drug testing

PZQ (kindly provided by Merck, Germany), OXM (Cat. No. PH002704), MFQ (Cat. No. M2319) and ART (Cat. No. A9361, Sigma-Aldrich, Germany) were dissolved in DMSO depending on drug solubility (PZQ 10 mg/ml, OXM 5 mg/ml, MFQ 33.3 mg/ml, ART 10 mg/ml) and stored at 4°C until use. NTS were cultured in HM supplemented with (SkS, LuS and LiS) or without (SkS and LuS) 20% HSe. To test the drug sensitivity of the distinct developmental stages, we incubated SkS (24-hour-old NTS), LuS (7-day-old NTS) and LiS (6-week-old NTS) parasites with PZQ, OXM, MFQ and ART at different concentrations (1, 10, 100 μg/ml). Before the addition of the drugs, a medium exchange was performed. For each condition experiments were performed in triplicates. Scoring was performed before (0 h) and 3, 24, 48, 72 and 168 h after treatment (a.t.).

### Statistics

For statistical analysis of the experiments to determine the optimal culture medium as well as the HSe concentration, a Kruskal-Wallis test was performed on day 1 and 3, week 1, 4 and 8 p.t. and if significant (p ≤ 0.05), the data was further analyzed by employing Mann-Whitney U tests comparing pure medium with serum-supplemented medium followed by a Bonferroni correction. For the statistical analysis of the experiment comparing HM to Basch medium 169, testing was performed in the same manner except that Mann-Whitney U testing was used to compare HM to Basch medium 169 for each of the shown conditions. For statistical analysis of the drug treatment experiments, Mann-Whitney U testing was performed to determine statistically significant differences between the DMSO control and the treated groups. Statistical testing was done with IBM SPSS Statistics 24 (IBM). Graphs were made with PRISM 5 (GraphPad).

## Results

### Serum- and cell-free medium ensures a high viability of *S*. *mansoni* NTS in long-term culture

**Fig 1:**
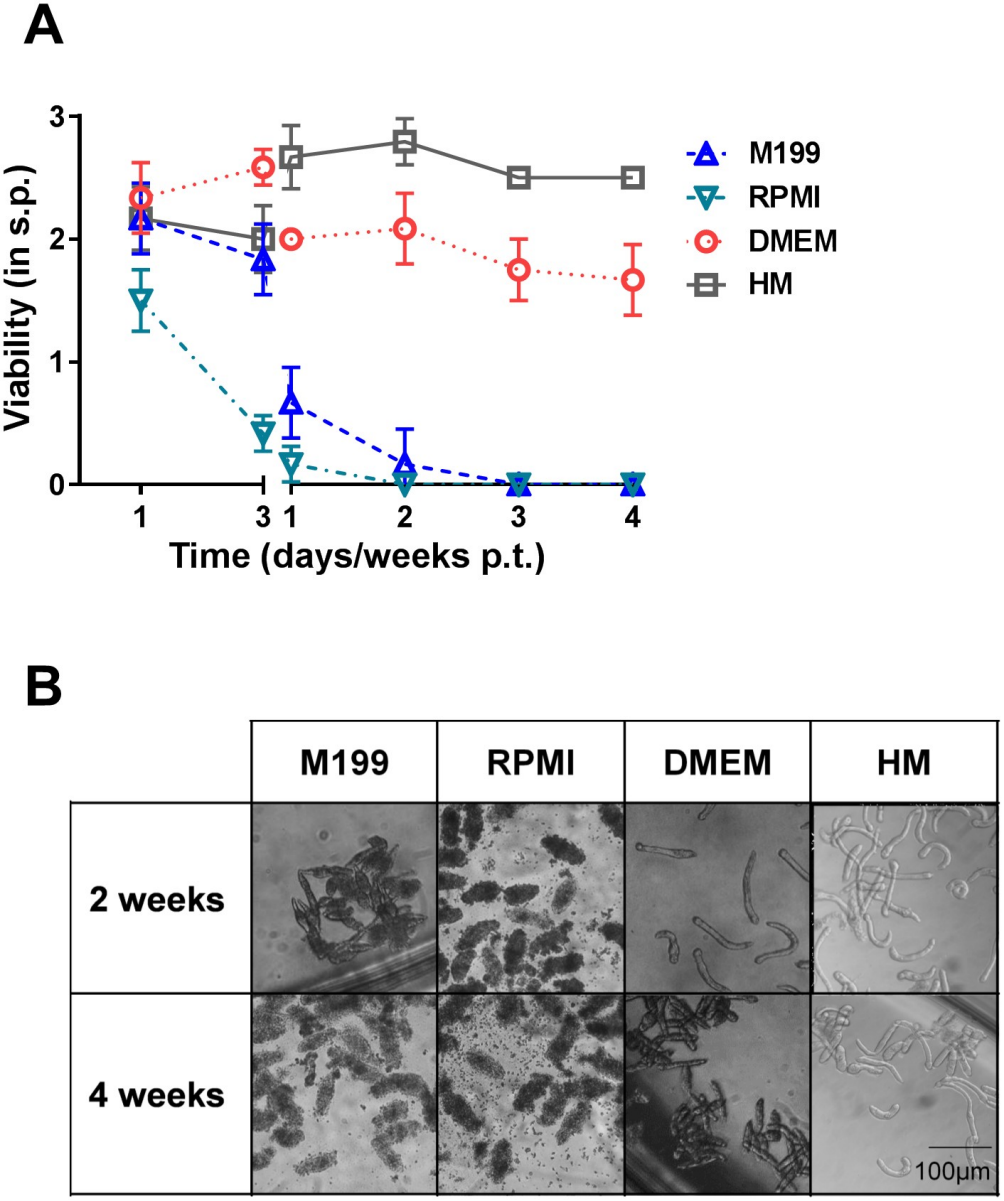
Culture media selectively ensure long-term viability of *S*. *mansoni* NTS without serum or cell supplementation. NTS were cultured in HM, M199, DMEM or RPMI supplemented with 200 U/ml Penicillin and 200 μg/ml Streptomycin for 4 weeks. (A) The viability of the parasites was scored at the indicated time points and (B) the morphology was observed under light microscopy. Photomicrographs were taken 2 and 4 weeks p.t. Scale bar applies to all shown images. Results are representative of at least three independent experiments. Each data point is shown as mean ± SD of at least three biological replicates. p.t., post-transformation; s.p., score points; HM, HybridoMed Diff 1000.

To generate and maintain NTS under serum- and cell-free conditions, we cultivated NTS immediately following transformation in different highly standardized and commercially available culture media such as HM, DMEM, RPMI and M199 (Fig 1A and 1B) supplemented only with 200 U/ml penicillin and 200 μg/ml streptomycin. Over a period of four weeks, regular visual viability scoring of the parasites was performed following defined criteria adapted from previously published works [27] (S1 Fig). In DMEM, NTS survived well for at least four weeks. Their viability peaked on day 3 p.t., (2.6 ± 0.1), characterized by an increased motility and hardly any granularity. Then viability declined slightly (2.0 ± 0.0) due to the death of single NTS and slight internal granulation on day 7 after which the viability stabilized throughout the remainder of the experiment (Fig 1A and 1B). In HM, NTS initially scored (2.0 ± 0.3) on day 3 p.t., slightly lower compared to those in DMEM. However, from one week p.t. onwards, NTS in HM had already surpassed those kept in DMEM in the viability scoring (2.7 ± 0.3) due to the absence of granularity and increase of regular, steady movement. NTS in HM stayed viable throughout the experiment (four weeks) (Fig 1A and 1B). Compared to these two media (HM and DMEM), M199 and RPMI supported viability of NTS rather poorly. In M199, viability already started to drop (1.8 ± 0.3) on the third day p.t., characterized by the majority of the NTS being heavily damaged or already dead. By week 3 p.t., all NTS had died (Fig 1A and 1B). In RPMI, the viability declined even faster with the majority of the NTS already heavily damaged or dead on day 3 p.t. and the death of all NTS by week 2 p.t. (Fig 1A and 1B). In all conditions, NTS were initially oval shaped on day 1 to day 3, resembling the SkS. Skin-stage schistosomula were characterized by a stumpy looking oval outline with regular elongations and contractions either straight or to the side and were 105.7 ± 7.8 μm in length (in HM) (S2 Fig). By one week p.t., the surviving NTS had developed LuS characteristics. LuS NTS were characterized by a more elongated and slender form with an increased activity compared with the SkS. The parasites’ movements were still characterized by regular elongation and contraction in changing directions with an average length of 184.4 ± 45.5 μm (Fig 1B and S2 Fig) [31]. In all media, however, development of surviving NTS halted after the first week of culture and, therefore, remained at the LuS stage for the remainder of the experiment. Dead NTS, in M199 or RPMI medium, displayed massive granulation internally and an irregular outer tegument. In contrast, healthy NTS, in HM and DMEM, were elongated and slender with a clear interior and a well-contrasted outline (Fig 1B). Taking all of this into consideration, HM is the most suitable medium for long-term culture of LuS NTS without any serum or cell supplementation; however, development under these conditions is halted in the LuS.

### HSe enhances NTS viability *in vitro* and induces development past the LuS up to late LiS parasites

**Fig 2:**
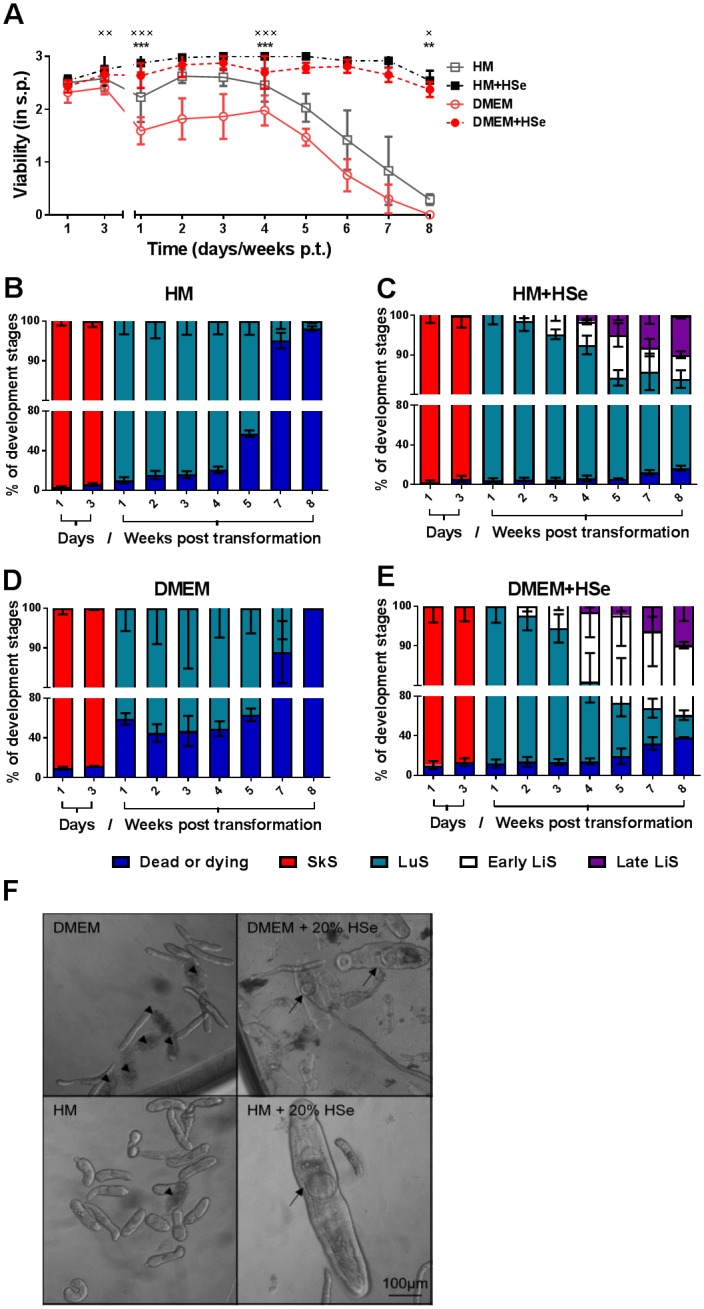
Presence of human serum *in vitro* enhances NTS viability and induces worm development past the LuS. NTS were cultured in HM and DMEM supplemented with 200 U/ml Penicillin and 200 μg/ml Streptomycin with or without additional supplementation of 20% HSe. (A) Viability scoring was performed at the indicated time points. The percentage of developmental stages in culture with either (B) HM, (C) HM + 20% HSe, (D) DMEM or (E) DMEM + 20% HSe was calculated for the indicated time points by bright field microscopy. (F) Representative photomicrographs were taken on day 35 post-transformation. Scale bar applies to all shown pictures. Arrowheads indicate dead NTS. Arrows indicate early and late LiS. Each data point is shown as a mean ± SD of pooled data from three independent experiments with three biological replicates each. * *p* ≤ 0.05, ** p ≤ 0.01, *** p ≤ 0.001 comparing HM with HM + 20% HSe, × p ≤ 0.05, ×× ≤ 0.01, ××× ≤ 0.001 comparing DMEM with DMEM + 20%. HSe, human serum; SkS, skin stage; LuS, lung stage; LiS, liver stage; s.p., score points; p.t., post-transformation.

To test whether, in the absence of any cell supplementation, LuS NTS could develop further into the LiS by adding serum of the most important definite host, namely the human [2, 32], we supplemented HM and DMEM with 20% HSe. In both serum-free controls, we observed a decline in viability after the fourth week of observation as before (Fig 2A). More specifically, in HM and DMEM, all SkS that survived, developed to the LuS by the first week p.t. Until the fourth week p.t., around 49% (48.7 ± 4.2 dead out of 99.7 ± 10.6 total parasite count/well) of the NTS died in DMEM compared to only around 21% (39.0 ± 9.0 dead out of 185.7 ± 14.0 total) in HM. Following the fourth week p.t., a steady increase in dead NTS was present in both conditions (Fig 2B and 2D). However, the addition of 20% HSe to the culture halted the loss of viability and greatly reduced NTS-death within the first 4 weeks of transformation. Interestingly, the next developmental stage, the early LiS started to develop from 2 weeks p.t., as evident by the further increase in size (growing wide and stumpy rather than in length) and the gut becoming clearly visible under the light microscope. By week 4 p.t., the parasite had developed further into the late LiS, characterized by clearly visible ventral and oral suckers and elongation of the aboral part of the parasite’s body. Its activity could be either restricted to the oral or aboral part of the body or could encompass the entire worm. This stage was characterized by a steady increase in length (1289.6 ± 247.0 μm) after 6 weeks of culture (S2 Fig). The time point of this development was the same for HM and DMEM. Even though the percentage of the early LiS in DMEM (29.4% or 34.0 ± 2.8 out of 115.5 ± 6.4 total) was higher than that in HM (6.1% or 9.3 ± 1.2 out of 153.0 ± 20.0), the percentage of the late LiS was comparable between DMEM (11.5 ± 5.0 out of 115.5 ± 6.4 total) and HM (15.3 ± 3.2 out of 153.0 ± 20.0) with around 10% having developed after 8 weeks of incubation (Fig 2C and 2E). Importantly even though the initial time point for the occurrence of the late LiS in HM and DMEM was the same, the overall growth rate of the parasites in HM supplemented with HSe was increased compared with that in DMEM since the late LiS in HM (week 4 and after) grew to be remarkably bigger (Fig 2F, arrows). Strikingly, despite the addition of HSe, a larger number (~ 40%) of NTS had died in DMEM by 8 weeks (Fig 2F) as compared to HM, where only ~10% of NTS were dead.

### Supplementation of HSe increases development to late liver stage in HM when compared to Basch medium 169

**Fig 3:**
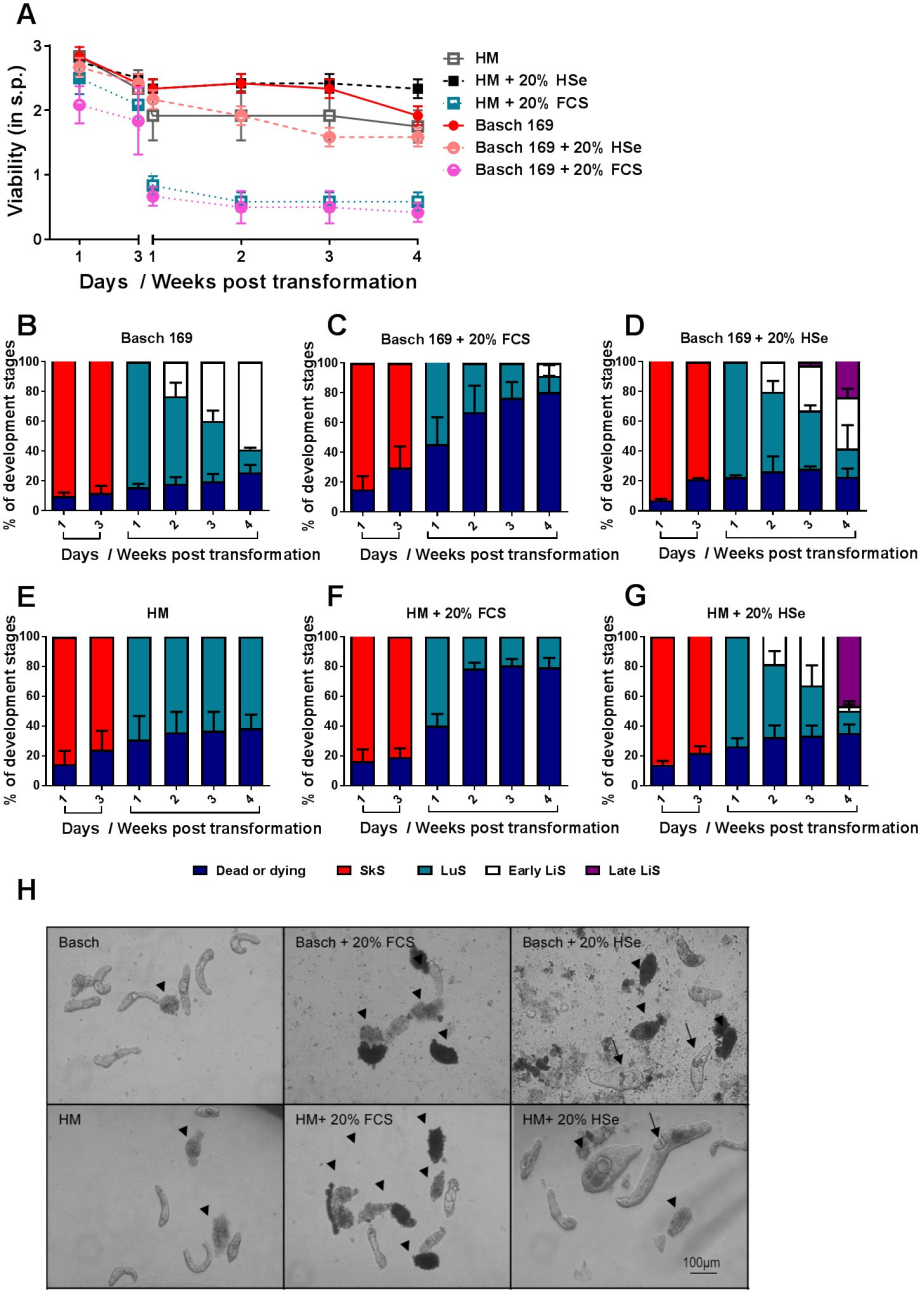
HSe supplemented HM increased the development to late liver stage compared to Basch medium 169. NTS of *Schistosoma mansoni* (NMRI strain) were cultured in HM and Basch-Medium 169 supplemented with 200 U/ml Penicillin and 200 μg/ml Streptomycin as well as additional supplementation of either 20% FCS or 20% HSe. (A) Viability scoring was performed at the indicated time points. The percentage of developmental stages in culture with (B) Basch medium 169 supplemented with (C) 20% FCS or (D) 20% HSe as well as (E) HM supplemented with (F) 20% FCS or (G) 20% HSe were calculated per well for the indicated time points by bright field microscopy. (H) Representative photomicrographs were taken on day 28 p.t. Scale bar applies to all images shown. Arrowheads indicate dead NTS. Arrows indicate early and late LiS. Each data point is shown as a mean ± SD of an experiment with three biological replicates each. FCS, fetal calf serum; HSe, human serum; SkS, skin stage; LuS, lung stage; LiS, liver stage; s.p., score points; p.t., post-transformation.

Since one of the media currently considered to be the “gold standard” to raise advanced developmental stages is Basch medium 169, we cultured NTS (derived from the *S*. *mansoni* NMRI strain) with Basch medium 169 and HM medium without further serum supplementation. Interestingly, NTS raised in HM again did not develop past the LuS, whereas in Basch medium 169, the development to the early LiS stage was observed starting from week two onwards. Furthermore, in Basch medium 169 the viability score (2.3 ± 0.1 at week 3 p.t.) was around 0.5 score points higher than in HM (1.9 ± 0.4 at week 3 p.t.) for the first 3 weeks of the experiment. This was followed by a slight drop in viability in Basch medium 169 in the fourth week with a final score of 1.9 ± 0.1 for Basch medium 169 and 1.8 ± 0.3 for HM. Upon addition of FCS, the currently most commonly used serum supplement in *in vitro* culture of *S*. *mansoni* [33–35], we could observe a drastic decline in viability for both media within the first week after transformation starting after the first three days of culture (0.8 ± 0.1 in HM and 0.7 ± 0.1 in Basch medium 169) (Fig 3A). In Basch medium 169 supplemented with 20% FCS the rate of development was reduced and delayed with only 8.7% early LiS by week 4 compared to 58.9% in unsupplemented medium. (Fig 3B and 3C). FCS supplementation to HM lead to an increase in dead NTS (from 38.7% to 79.4% dead parasites on week 4 p.t.) and was unable to promote development past the LuS. (Fig 3E and 3F). The addition of HSe to Basch medium did not further increase the viability score in contrast to the addition of HSe to HM which resulted in an overall increase of ~0.5 viability score points (2.3 ± 0.1 score points on week 4 p.t.) (Fig 3A). HSe was able to promote development to the late LiS in both media, and the first late LiS were observed 3 weeks p.t. in Basch medium 169 and 4 weeks p.t. in HM. However, the percentage of developing late LiS was much higher in HM (46.7%) compared to Basch-medium 169 (23.9%) (Fig 3D and 3G) indicating that addition of HSe to HM had a more pronounced effect on promoting larval development towards juvenile worms.

### Human serum increases the efficiency of late LiS worm generation in a concentration-dependent manner

**Fig 4:**
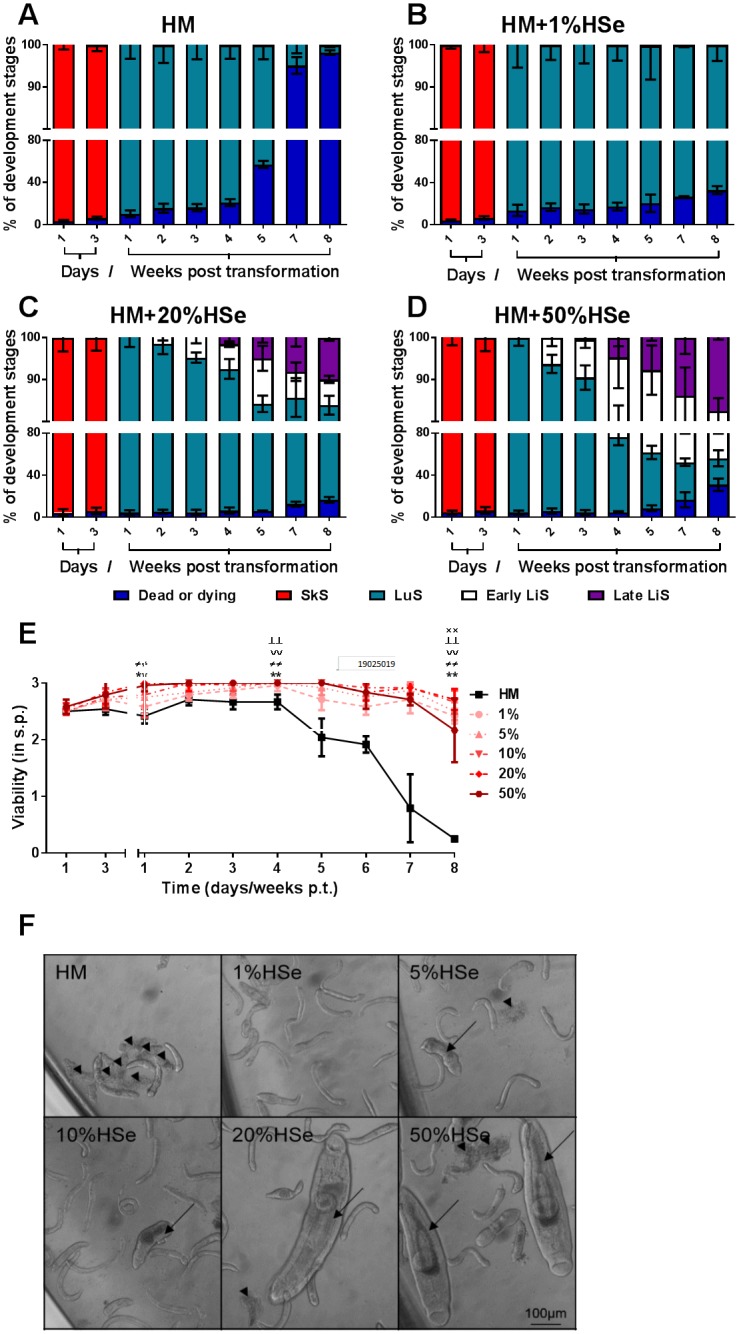
Generation of late LiS worm in medium supplemented with human serum increases in a concentration-dependent manner. NTS were cultured in HM supplemented with 200 U/ml Penicillin and 200 μg/ml Streptomycin as well as 1%, 5%, 10%, 20% and 50% of HSe. The percentages of the developmental stages as well as dead parasites in culture with (A) HM alone or supplemented with (B) 1%, (C) 20% and (D) 50% HSe were calculated at indicated time points and (E) their viability was scored during bright field microscopy. (F) Representative photomicrographs were taken on day 35 post transformation. Scale bar applies to all pictures shown. Arrowheads indicate dead NTS. Arrows indicate LiS (early LiS in 5% and 10% HSe) and late liver stage (in 20% and 50% HSe). Results are pooled from three independent experiments with at least three biological replicates each. ×× p ≤ 0.01 comparing HM vs. 1% HSe; ┴┴ p ≤ 0.01 comparing HM vs. 5% HSe; ∨∨ p ≤ 0.01 comparing HM vs. 10% HSe; ≠≠ p ≤ 0.01 comparing HM vs. 20% HSe; ** comparing p ≤ 0.01 HM vs. 50% HSe. HSe, human serum; s.p., score points; p.t., post-transformation; SkS, skin stage; LuS, lung stage; LiS, liver stage.

Next, we investigated whether HSe induced development of advanced larval stages of *S*. *mansoni* increases in a concentration-dependent manner. Therefore, we cultured NTS in HM supplemented with 1, 5, 10, 20 or 50% of HSe. Even at the lowest concentration (1%), HSe successfully prevented the death of NTS which otherwise started to occur in the 5^th^ week p.t. (Fig 4A) and survival rates remained steadily above 67% (49.0 ± 7.4 dead in 151.7 ± 15.3 total) throughout the experiments (8 weeks) in all HSe-supplemented conditions (Fig 4B–4D). Surprisingly, however, at a concentration of 50% HSe, the survival rate (69.1% or 44.7 ± 10.1 dead out of 144.7 ± 21.2 total) as well as viability (2.2 ± 0.6) started to decline around week 8 (Fig 4D and 4E). Nevertheless, the final viability score and survival rate was still higher than in the control (viability score of 0.3 ± 0.0 and survival rate of 1.8% or 160.3 ± 7.8 dead out of 163.3 ± 8.5 total) (Fig 4A and 4E). However, development of NTS up to early and late LiS was only observed at higher concentrations (single early LiS starting in 5% HSe and late in 20% HSe) of HSe (Fig 4C, 4D and 4F). Early LiS development could be observed starting 2 weeks p.t. in all concentrations but their number LiS increased concentration-dependently. The first parasites in the late LiS were detected at 4 weeks p.t. in 20% HSe and single worms even one week earlier in 50% HSe. The overall percentage of late LiS parasites increased with rising serum concentrations, and, finally, around 10% LiS had developed (15.3 ± 3.2 in 153.0 ± 20.0 total parasite count/well) in 20% HSe compared to approx. 18% (25.3 ± 4.2 in 144.7 ± 21.2 total) in 50% HSe-supplemented HM (Fig 4C and 4D). Since viability decreased in 50% HSe-supplemented medium after the 7^th^ week p.t. and sufficient numbers (13–19 late LiS/well) of late LiS worms were generated at 20% HSe [26, 28], we decided to use 20% HSe supplementation for the generation of parasites for drug screening experiments.

### Drugs with known anti-schistosomal properties kill schistosomula in a stage-dependent manner

**Fig 5:**
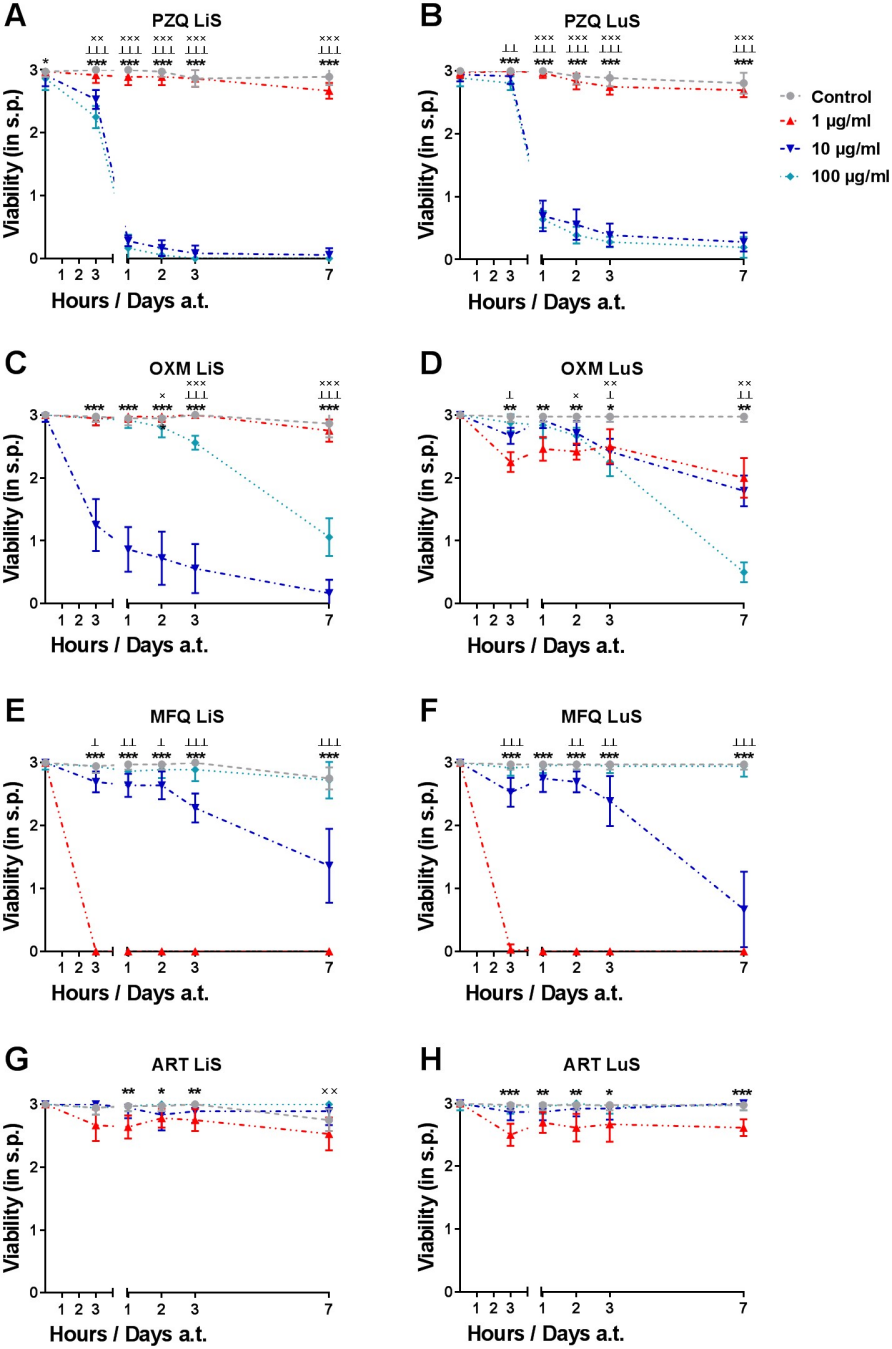
Efficient drug screening of advanced larval stages of *S*. *mansoni* generated in human serum. NTS were cultured in HM supplemented with 200 U/ml Penicillin and 200 μg/ml Streptomycin and 20% HSe for 1 week to generate LuS (B, D, F and H) and 6 weeks to obtain LiS (A, C, E, G) schistosomula. (A, B) Praziquantel (PZQ), (C, D) Oxamniquine (OXM), (E, F) Mefloquine (MFQ) and (G, H) Artemether (ART) were dissolved in DMSO and added at indicated concentrations for the entire duration of the experiment. 1% DMSO in culture medium served as control. Viability was scored before treatment (0 h) and 3 hours, 1, 2, 3 and 7 days a.t. Each data point is shown as a mean ± SD of three independent experiments with at least three biological replicates each. ××× p ≤ 0.001, ×× p ≤ 0.01, × p ≤ 0.05 comparing control with 1 μg/ml drug; ┴┴┴ p ≤ 0.001 ┴┴ p ≤ 0.01 ┴ p ≤ 0.05 control with 10 μg/ml drug; ***p ≤ 0.001, **p ≤ 0.01 *p ≤ 0.05 control with 100 μg/ml drug. LuS, lung stage; LiS, liver stage; s.p., score points; a.t., after treatment.

To test the different stages (SkS, LuS and late LiS) generated with the new method in HM and 20% HSe supplementation for the *in vitro* drug testing, we chose drugs with known anti-schistosomal properties [28, 36]. Late LiS (6-week-old schistosomula) (Fig 5A, 5C, 5E and 5G), LuS (7-day-old NTS) (Fig 5B, 5D, 5F and 5H) and SkS (24-hour-old NTS) (S3A–S3D Fig) NTS were cultured in the presence or absence of different concentrations (100, 10 and 1 μg/ml) of PZQ (Fig 5A and 5B and S3A Fig), OXM (Fig 5C and 5D and S3B Fig), MFQ (Fig 5E and 5F and S3C Fig) or ART (Fig 5G and 5H and S3D Fig) and assessed by regular viability scoring.

**Fig 6:**
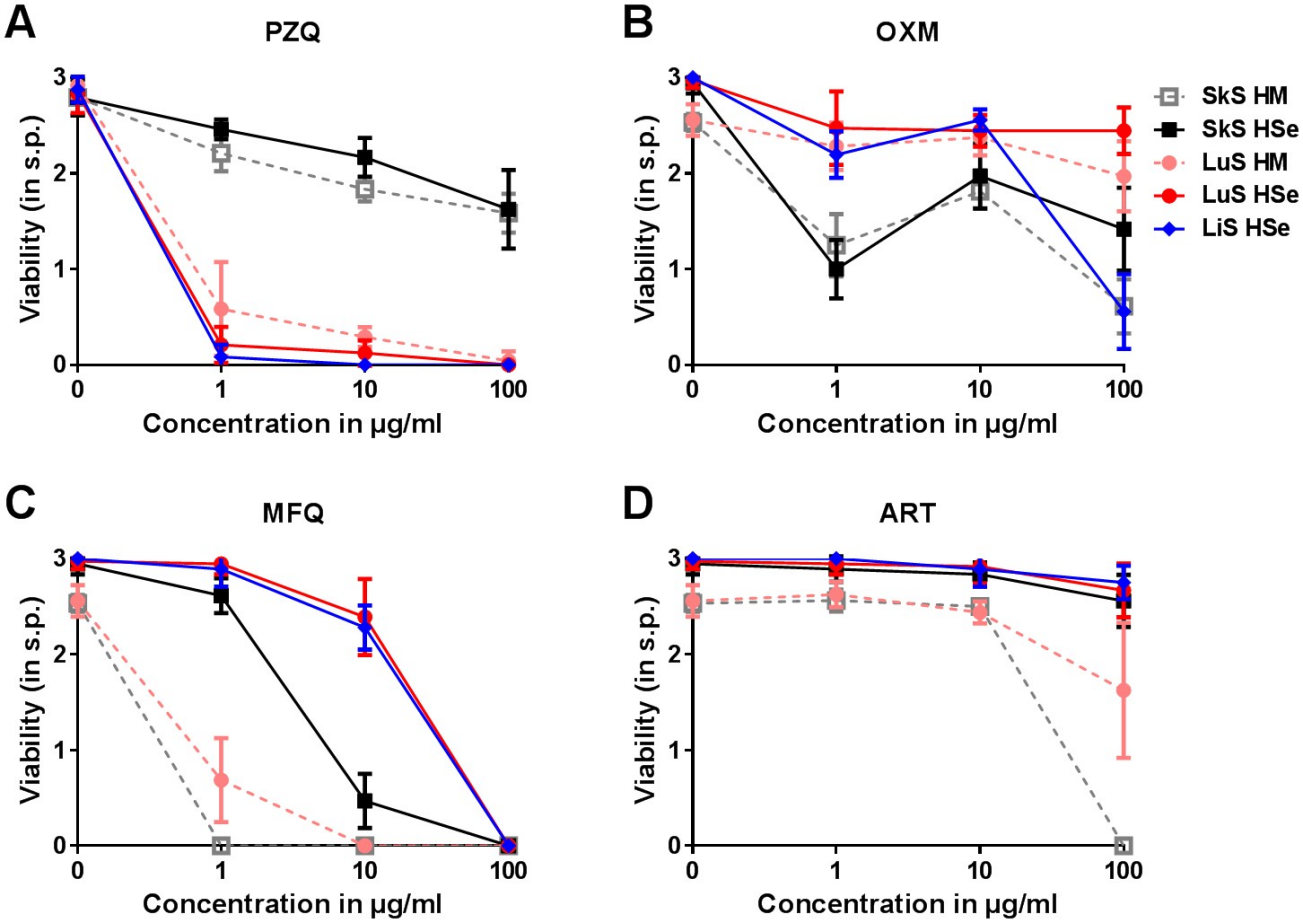
Drug sensitivity is dependent on the developmental stage of *S*. *mansoni* larvae generated *in vitro*. NTS were cultured and matured in HM supplemented with 200 U/ml Penicillin and 200 μg/ml Streptomycin only or additionally supplemented with 20% HSe. 100, 10 and 1 μg/ml of (A) praziquantel, (B) oxamniquine, (C) mefloquine and (D) artemether were then added to the culture 1 day p.t. to test the SkS, 7 days p.t. to test the LuS and 6 weeks p.t. to test the LiS. Viability was scored 72h a.t. Each data point is shown as a mean ± SD of three independent experiments with at least three biological replicates each. ××× p ≤ 0.001, ×× p ≤ 0.01, × p ≤ 0.05 comparing control with 1 μg/ml drug; ┴┴┴ p ≤ 0.001, ┴┴ p ≤ 0.01, ┴ p ≤ 0.05 comparing control with 10 μg/ml drug; ***p ≤ 0.001, **p ≤ 0.01 *p ≤ 0.05 control with 100 μg/ml drug. SkS, skin stage; LuS, lung stage; LiS, liver stage; PZQ, praziquantel; OXM, oxamniquine; MFQ, mefloquine; ART, artemether; s.p., score points.

PZQ treatment was detrimental to LiS and LuS at all concentrations, which was clearly evident from the drop in viability as soon as 3h a.t. and the death of almost all parasites on day 1 a.t. (Fig 5A and 5B), characterized by contracted parasites, damaged tegument and no detectable motility (S4 Fig). No such pronounced effect of PZQ was observed on SkS, however, only high concentrations of PZQ slightly reduced the observed viability starting at 3h a.t., but NTS viability then stabilized for the remaining part of the experiment (S3A Fig). In serum-free conditions, PZQ activity was comparable to serum-supplemented HM as observed on day 3 a.t. At that time point, the susceptibility of the LiS and LuS to PZQ compared with that of the SkS. Serum-free conditions did not alter the activity profile of PZQ on the SkS or LuS (Fig 6A). OXM and MFQ both had similar effects on the LiS at high concentrations. In particular, MFQ had a clear and strong anti-schistosomal activity at 100 μg/ml. In contrast to MFQ, OXM was also potent at 1 μg/ml. even more so than at 10 μg/ml (Fig 5C and 5E). MFQ showed similar activity on the SkS and LuS compared to the LiS (Fig 5E and 5F and S3C Fig). Overall, OXM was potent in reducing the viability of the parasites in the SkS at all tested concentrations. Even though the LuS was the least susceptible stage to OXM, a reduction in the viability could still be observed which was most prominent at 1 μg/ml (Fig 5D). The stage-dependent vulnerability of the schistosomula was observed quite well on day 3 a.t. It is worth mentioning that LuS and SkS NTS were slightly more susceptible to OXM in serum-free culture in the concentration of 100 μg/ml. Also, MFQ exerted an increased activity against both SkS and LuS NTS in serum-free compared to HSe-supplemented culture (Fig 6B and 6C). Closer and careful observations revealed that the highest concentration of OXM induced a hyperactive state directly following the addition of the drug at all stages, which was followed by heavy granulation and tegumental damage in the SkS and LiS already starting on day 1 a.t. and, in the LuS, slightly delayed starting one week a.t. Morphologically, MFQ caused almost instantaneous contraction as well as heavy granulation with a blurred, disintegrated outline of the parasite at 100 μg/ml (S4 Fig). ART did not show any effect on viability or a morphological alteration in LiS or LuS (Fig 5G and 5H and S4 Fig), but a slight reduction in viability of the SkS at 100 μg/ml (S3D Fig). On day 3 a.t., we could not detect a drop in viability or any morphological changes in any of the tested stages in HSe-supplemented medium (S4 Fig) or in serum-free medium. However, in the serum-free medium we could detect a clear schistosomicidal effect at 100 μg/ml, with death following an initial paralysis of the parasites. However, upon addition of the drug to serum free-culture, the previously dissolved drug precipitated to a small extent (Fig 6D). Taken together, we could show the varying stage-dependent activity profiles of selected compounds already known for their anti-schistosomal properties and no schistosomicidal activity for artemether.

## Discussion

In the London Declaration of 2012, the world, represented by partners in governments, academia, NGOs, pharmaceutical companies and more, committed itself to accelerate the control, elimination and eradication of 10 NTDs by 2020 [37]. Despite successes in several areas, progress towards the elimination of schistosomiasis has remained rather limited [38]. Reasons for this lack of progress range from challenges in schistosome vector control to limited developments in drug discovery programs. Rather than developing new compounds, the scarce resources are focused towards drug repurposing. The limited interest of pharmaceutical companies in researching entirely new compounds is due to the resource- and time-consuming process to get an approval by the health authorities reviewed in 2014 by Panic et al. [39]. Another limitation in *in vitro* screening used for testing compound libraries is the availability of *in vitro*-generated advanced stage parasites. Therefore, current *in vitro* compound screenings of *S*. *mansoni* still mostly focus on SkS schistosomula observed for up to 72 h after treatment for initial hit identification or on adult worms retrieved from infected mice for hit confirmation [14, 40, 41]. This focus leaves a “blind spot” for drug efficacy toward intermediate developmental stages of the parasite. The value of monitoring drug efficacy for initial hit identification at all consecutive stages of parasite development in parallel is clearly illustrated by the stage-specific efficacy of PZQ, which shows decreased efficiency between 1 day and 5 weeks of development with minimal worm burden reduction at the age of 4 weeks *in vivo* [42, 43], but still represents the most effective and widely used schistosomicidal drug available [44]. Studies on *ex vivo* harvested LuS schistosomula sometimes manage to bridge this gap, but are challenging and require a living host (invoking practical as well as ethical aspects) [45].

*In vitro S*. *mansoni* culture systems obviously can circumvent some of these challenges and allow for the continual observation of consecutive larval stages. *In vitro* transformation and culture techniques have first been established in the 1970s and 1980s [17, 46] and since then different culture media have rarely been evaluated [47]. Until today, those techniques rely on the presence of serum (mainly FCS) for early larval stages [48, 49] and the addition of human blood cells to generate advanced larval and juvenile worm stages [16, 50]. Viability scoring relies on visual assessment of the larvae in culture by bright field microscopy, which still is the gold standard for drug efficacy tests [27, 28]. While the method has its merits and can be highly effective for dedicated drug efficacy testing, it is very labor intensive. At the same time, microphotographic-based automated analysis [51] using algorithms is complicated by the presence of large numbers of RBCs and/or PBMCs that overlay or co-localize with larvae, making reliable assessment of tegument damage, for example, something for the “trained eye” only. Moreover, the repeated addition of fresh cells from human donors is a factor that is difficult or impossible to fully standardize.

In this study, we generated a novel cell-free *in vitro* assay that allows the development and long-term observation of *S*. *mansoni* larvae. Mechanically transformed NTS [25, 28] were successfully cultured in a serum- and cell-free culture medium for up to four weeks, allowing for their development to LuS schistosomula but not further. Supplementation with 20% FCS decreased the viability after the third day of cultivation and the developmental block persisted throughout the period of cultivation. Most recently published in vitro NTS assays that have reported the use of FCS as supplement in either Medium 169 or Medium 199 do not score viability after 3 days of culture [33–35]. Thus, the relative loss in viability in FCS supplemented HM after three days needs to be explored further since batch differences in FCS are a well-known phenomenom in cell culture assays and could explain our observations as well. However, the addition of HSe to the culture broke the developmental block at the LuS and propelled development to LiS worms, and survival for at least eight weeks. Thus, this cell-free culture for advanced stage *S*. *mansoni* represents important progress in existing tools that rely on the addition of “non-standardized” human blood cells [16]. Instead, the commercially available HM, normally used to cultivate hybridomas and other cell lines, is highly standardized, supplemented with transferrin, insulin, bovine serum albumin (BSA)/oleic acid complex, absorbable amino acids, D-glucose, vitamins and minerals. The presence of albumin and insulin may be critical for the prolonged survival (≥ 4 weeks) of parasites under serum-free conditions, and indeed, earlier findings showed that schistosomula can ingest and digest albumin and IgG *in vitro*, utilizing them as nutrient sources [52]. In addition, it was shown that *S*. *mansoni* has two insulin receptors (SmIR1 and SmIR2), essential for the survival of the pathogen *in vitro* as well as *in vivo* [53]. Nevertheless, and in contrast to the natural development *in vivo*, NTS development halted in serum-free insulin-containing media at a stage that, in terms of size and morphology, closely resembles that found in the lung vasculature [21]. This indicates that other factors than insulin or albumin contribute to the further development beyond the lung-stage. This is possibly not so surprising considering that the parasite is blood-dwelling and therefore permanently surrounded by the blood components. Nevertheless, FCS which is widely used in *in vitro* culture [33–35] could not see development past the LuS. Cattle, in contrast to humans, is an atypical definitive host for *S*. *mansoni*, and nevertheless an important alternative definitive host [2, 32, 54]. HSe did not only improve overall viability but also induced development of NTS to LiS parasites in a concentration-dependent manner. When compared to ‘gold standard’ Basch medium 169, which is a non-commercial mixture of 15 components which needs to be freshly prepared before use [17], HM supplemented with HSe yielded comparable viability scores but increased the development of LiS schistosomes *in vitro*. Furthermore, we observed a difference in the rate of development between two strains of *S*. *mansoni*, whereby late liver stage parasites were detected in a higher proportion in the NMRI compared to the Brazilian strain starting from the 4^th^ week p.t. (Fig 3A and 3B). Differences in the dynamics of development of several *S*. *mansoni* strains within a host have been previously demonstrated using an *in vivo* mouse model [55, 56].

Juvenile worms could be maintained for extended periods of time (more than a year although no pairing or egg production could be observed) through the addition of HSe to HM alone in contrast to non-supplemented controls. However, concentration-dependent advances in stage development (in 50% HSe) came at the price of increased larval mortality at seven weeks and onwards. This may be explained by the increased numbers of parasites reaching the LiS, their increased size and corresponding increased rate in medium nutrient depletion. This would be in line with the fact that the parasite’s feeding and cell divisions are limited, both indicators of low metabolism rates until it reaches the LiS about 15 days after infection *in vivo* [21]. We found supplementation with 20% HSe to provide an optimum between HSe consumption and frequency of medium changes, generating a reasonable number of late LiS worms for further studies. This allows the incorporation of all developmental stages in initial hit identification strategies for future drug screenings, making it possible to identify compounds with an activity profile predominantly targeting lung to liver stage parasites. Such drugs would probably otherwise be missed. Taken together, we show that in contrast to cercariae that are susceptible to the complement system present in serum [57], schistosomula not only tolerate the presence of HSe but require it to progress to late larval stages within their definitive host, the human.

Another aim of this study was to investigate the suitability of this method to screen compound libraries for schistosomicidal activity against, in particular, advanced stages of *S*. *mansoni* in addition to the mostly used early stages [28, 51]. NTS showed comparable stage-specific susceptibility to four drugs known to possess anti-schistosomal properties as seen previously with other *in vitro* assays [16, 28], confirming the validity of our novel culture method. For example, PZQ, undoubtedly the most important tool to control schistosomiasis in the field, [16, 58] was described to have reduced efficacy starting 24h after infection and lasting until day 35 after infection when susceptibility is increasing again [42, 43, 59]. We could confirm this in our novel culture with the SkS (day 1) and LuS parasites (day 7) being less vulnerable compared to late LiS worms (day 42) which is in accordance with previous publications although to a slightly lesser extent. This could be attributed on the one hand to the difference in the media that were employed (e.g. M199, RPMI 1640 or DMEM as compared to HM) [33–35] and on the other hand to the difference in *S*. *mansoni* strains used to generate NTS for *in vitro* drug testing [28, 60, 61]. The substantial tegumental damage and anti-schistosomal activity in our assay induced by OXM, the drug used against *S*. *mansoni* before the discovery of PZQ [62, 63], was also comparable to that observed by others [16, 28]. Specifically, morphological changes of the tegument as well as schistosomicidal activity, which was more pronounced against LiS and SkS schistosomula at high concentrations, could be observed. The increased activity in 1 μg/ml compared with 10 μg/ml of OXM was surprising. A similar observation has been made in a previous study [28], but the reason for this is still unknown. MFQ, a well-known anti-malarial drug, that was shown to also be active against *S*. *mansoni in vivo* and *in vitro* [64], is thought to impact the heme detoxification as well as glycolysis in the parasite [65]. Interestingly, the stage-dependent activity profile of MFQ is different to that of PZQ and more potently targets NTS than mature worms [29], something we were able to confirm in our novel assay as well. ART is another anti-malarial drug that was previously shown to act against *S*. *mansoni* [36] and is thought to act via toxication by the hemin byproduct of the parasite’s digestion of hemoglobin. Indeed, in the absence of a cellular source of hemoglobin and comparable to our cell-free system, ART lost activity, supporting the notion that hemin is required for the efficacy of the drug [66]. In the absence of serum during early developmental stages, we found strongly enhanced schistosomicidal activity of MFQ, slightly increased potency of ART, but mostly unchanged activity of PZQ and OXM. Bioavailability of MFQ is known to be strongly dependent on binding to plasma proteins (which can be as high as 98% [67]), and for ART plasma-binding lies between 92–98%, in line with our observed increase in activity in serum-free cultures [68]. On the other hand, PZQ binds to a lesser extent to plasma proteins (~80%) in concentrations of 10–100 μg/ml and *in vitro* as low as 50% [69], something that is also reflected in the limited increase in toxicity we observed in non-serum supplemented cultures. Importantly, our assay allows researchers to continually observe toxicity effects and inhibition of maturation from the SkS (day 1–3 p.t.) parasite over LuS (from day 7 p.t.) and early LiS (from day 14 p.t.) to the late LiS (from day 28 p.t.) in settings ranging from compound or drug testing, but also to screen for natural factors from non-permissive hosts or, reversely, to identify growth-promoting compounds from the host-adapted, parasite friendly environment.

Taken together, since late LiS worms generated in our cell-free assay showed drug-specific phenotypes for all drugs except artemether and responded similarly to *ex vivo* harvested worms from infected laboratory animals [28], this assay has the potential to reduce the reliance on *in vivo* generated worms by replacing *ex vivo* harvested worms in the initial hit identification and to thereby reduce costs and labor for large-scale drug screening assays. We are, however, aware that hit confirmation in *ex vivo* harvested worms might remain a necessity and that further studies such as comparative gene-expression of *in vivo* and *in vitro* generated parasite stages are ultimately necessary to clarify the extent of similarity. Eventually, the independence from host blood cells facilitates the automated assessment of larval viability in large-scale assays, due to a lack of visual interference by host cells. In addition, the high level of standardization will allow researchers to investigate and identify components within HSe that are exploited by the parasite for its development in the dominant definite human host and thus define mechanisms that underlie the host-specificity of this parasite. Ultimately, such understanding will pave the way for the identification of new drug and vaccine targets.

## Supporting information

S1 Fig Detailed description of morphological parameters to determine the viability score points of NTS *in vitro*. Photomicrographs represent indicated viability score points, which results from the overall assessment of motility, morphology and granularity of all NTS per well. Photomicrographs were taken on day 7 post-transformation using a digital camera fitted to an inverted microscope. Scale bar applies to all shown pictures.
(TIF)

S2 Fig *In vitro* developmental stages from skin stage schistosomula to late LiS worms. NTS were cultured in Hybrido Med (HM) supplemented with 200 U/ml Penicillin and 200 μg/ml Streptomycin and 20% human serum (HSe). Photomicrographs were taken at indicated time points. For skin, lung and day 21 liver stage the scale bar displayed in d21 LiS applies, whereas the scale bar for d35 only applies to this picture.
(TIF)

S3 Fig *In vitro* drug testing on skin stage NTS. NTS were cultured in HM supplemented with 200 U/ml Penicillin and 200 μg/ml Streptomycin and 20% HSe. (A) PZQ, (B) OXM, (C) MFQ and (D) ART were dissolved in DMSO and added at 100, 10 and 1 μg/ml 24 h p.t. and the viability was scored at indicated time points. 1% DMSO in culture medium served as control. Each point is shown as a mean ± SD of three independent experiments with at least three biological replicates each ××× p≤0.001, ×× p≤0.01, × p≤0.05 ┴┴┴ comparing control with 1 μg/ml drug; p≤0.001 ┴┴ p≤0.01 ┴ p≤0.05 control with 10 μg/ml drug; ***p < 0.001, **p < 0.01 *p≤0.05 control with 100 μg/ml drug. s.p., score points; a.t., after treatment; PZQ, praziquantel; OXM, oxamniquine; MFQ, mefloquine; ART, artemether.
(TIF)

S4 Fig Drug-induced morphological changes in all developmental stages. NTS were cultured in HM supplemented with 200 U/ml Penicillin and 200 μg/ml Streptomycin and 20% HSe. PZQ, OXA, MFQ and ART were dissolved in DMSO and added for a final concentration of 100 μg/ml to the culture, HM supplemented with 1% DMSO served as control. Photomicrographs were taken 24h after drug treatment. Scale bar applies to all shown pictures. PZQ, praziquantel; OXM, oxamniquine; MFQ, mefloquine; ART, artemether.
(TIF)
