# Correction: A novel cell-free method to culture *Schistosoma mansoni* from cercariae to juvenile worm stages for in vitro drug testing

**DOI:** 10.1371/journal.pntd.0010513

**Published:** 2022-06-02

**Authors:** Sören Frahm, Anisuzzaman Anisuzzaman, Ulrich Fabien Prodjinotho, Nermina Vejzagić, Admar Verschoor, Clarissa Prazeres da Costa

After publication of this article, the authors noted errors in the labels, legend, and data reported in Fig 5. An updated version of Fig 5 and its legend are provided here, and the underlying data for results reported in Fig 5 are in S5 File.

**Fig 5:**
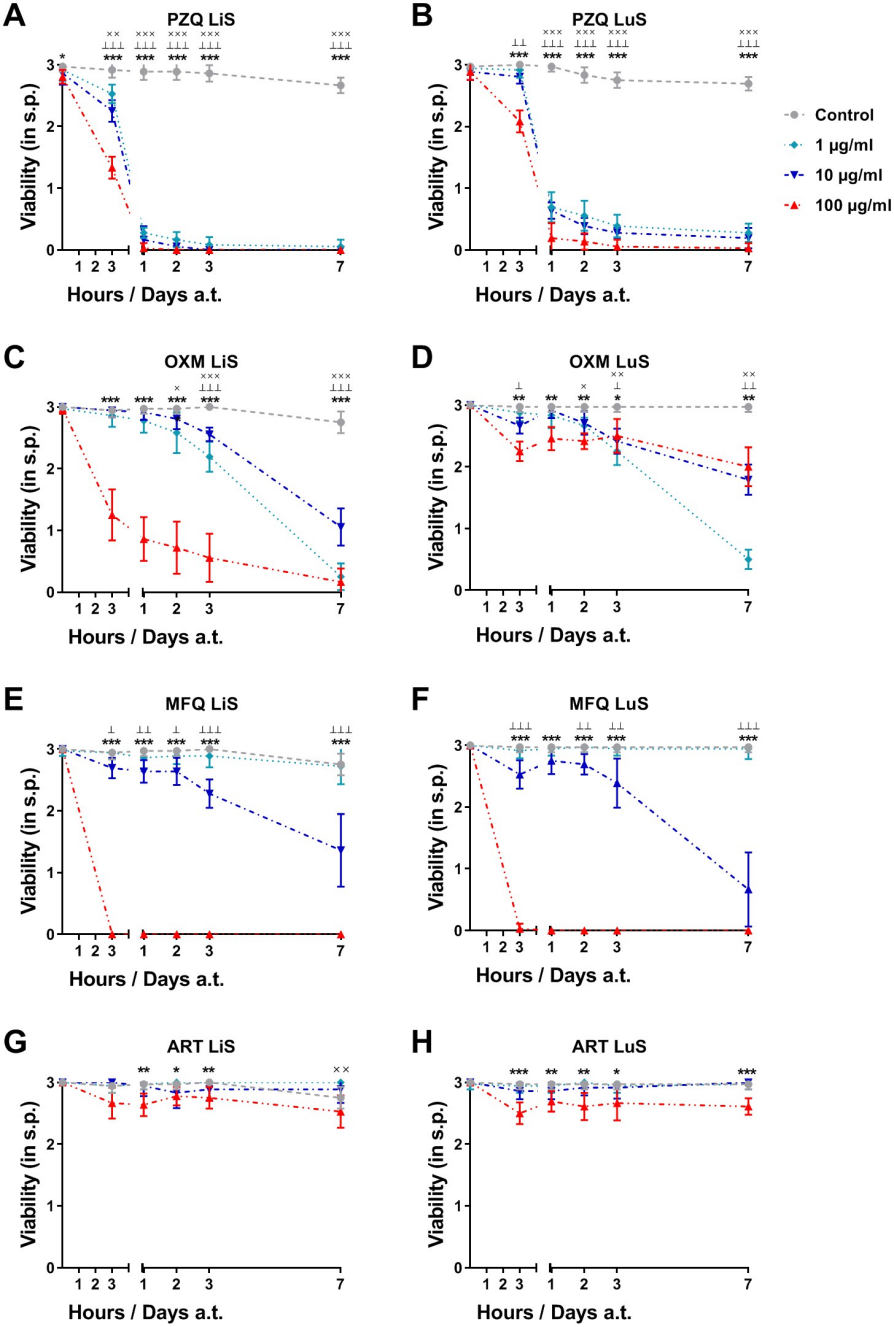
Efficient drug screening of advanced larval stages of *S*. *mansoni* generated in human serum. NTS were cultured in HM supplemented with 200 U/ml Penicillin and 200 μg/ml Streptomycin and 20% HSe for 1 week to generate LuS (B, D, F and H) and 6 weeks to obtain LiS (A, C, E, G) schistosomula. (A, B) Praziquantel (PZQ), (C, D) Oxamniquine (OXM), (E, F) Mefloquine (MFQ) and (G, H) Artemether (ART) were dissolved in DMSO and added at indicated concentrations for the entire duration of the experiment. 1% DMSO in culture medium served as control. Viability was scored before treatment (0 h) and 3 hours, 1, 2, 3 and 7 days a.t. Each data point is shown as a mean ± SD of three independent experiments with at least three biological replicates each. ××× p ≤ 0.001, ×× p ≤ 0.01, × p ≤ 0.05 comparing control with 1 μg/ml drug; ┴┴┴ p ≤ 0.001 ┴┴ p ≤ 0.01 ┴ p ≤ 0.05 control with 10 μg/ml drug; ***p ≤ 0.001, **p ≤ 0.01 *p ≤ 0.05 control with 100 μg/ml drug. LuS, lung stage; LiS, liver stage; s.p., score points; a.t., after treatment.

In addition, the underlying data were not included with the original published article, and are provided here in S1–S6 Files.

## Supporting information

S1 File Culture media selectively ensure long-term viability of *S*. *mansoni* NTS without serum or cell supplementation. NTS were cultured in HM, M199, DMEM or RPMI supplemented with 200 U/ml Penicillin and 200 μg/ml Streptomycin for 4 weeks. (A) The viability of the parasites was scored at the indicated time points and (B) the morphology was observed under light microscopy. Photomicrographs were taken 2 and 4 weeks p.t. Scale bar applies to all shown images. Results are representative of at least three independent experiments. Each data point is shown as mean ± SD of at least three biological replicates. p.t., post-transformation; s.p., score points; HM, HybridoMed Diff 1000.
(PZFX)

S2 File Presence of human serum *in vitro* enhances NTS viability and induces worm development past the LuS. NTS were cultured in HM and DMEM supplemented with 200 U/ml Penicillin and 200 μg/ml Streptomycin with or without additional supplementation of 20% HSe. (A) Viability scoring was performed at the indicated time points. The percentage of developmental stages in culture with either (B) HM, (C) HM + 20% HSe, (D) DMEM or (E) DMEM + 20% HSe was calculated for the indicated time points by bright field microscopy. (F) Representative photomicrographs were taken on day 35 post-transformation. Scale bar applies to all shown pictures. Arrowheads indicate dead NTS. Arrows indicate early and late LiS. Each data point is shown as a mean ± SD of pooled data from three independent experiments with three biological replicates each. * *p* ≤ 0.05, ** p ≤ 0.01, *** p ≤ 0.001 comparing HM with HM + 20% HSe, × p ≤ 0.05, ×× ≤ 0.01, ××× ≤ 0.001 comparing DMEM with DMEM + 20%. HSe, human serum; SkS, skin stage; LuS, lung stage; LiS, liver stage; s.p., score points; p.t., post-transformation.
(PZFX)

S3 File HSe supplemented HM increased the development to late liver stage compared to Basch medium 169. NTS of *Schistosoma mansoni* (NMRI strain) were cultured in HM and Basch-Medium 169 supplemented with 200 U/ml Penicillin and 200 μg/ml Streptomycin as well as additional supplementation of either 20% FCS or 20% HSe. (A) Viability scoring was performed at the indicated time points. The percentage of developmental stages in culture with (B) Basch medium 169 supplemented with (C) 20% FCS or (D) 20% HSe as well as (E) HM supplemented with (F) 20% FCS or (G) 20% HSe were calculated per well for the indicated time points by bright field microscopy. (H) Representative photomicrographs were taken on day 28 p.t. Scale bar applies to all images shown. Arrowheads indicate dead NTS. Arrows indicate early and late LiS. Each data point is shown as a mean ± SD of an experiment with three biological replicates each. FCS, fetal calf serum; HSe, human serum; SkS, skin stage; LuS, lung stage; LiS, liver stage; s.p., score points; p.t., post-transformation.
(PZFX)

S4 File Generation of late LiS worm in medium supplemented with human serum increases in a concentration-dependent manner. NTS were cultured in HM supplemented with 200 U/ml Penicillin and 200 μg/ml Streptomycin as well as 1%, 5%, 10%, 20% and 50% of HSe. The percentages of the developmental stages as well as dead parasites in culture with (A) HM alone or supplemented with (B) 1%, (C) 20% and (D) 50% HSe were calculated at indicated time points and (E) their viability was scored during bright field microscopy. (F) Representative photomicrographs were taken on day 35 post transformation. Scale bar applies to all pictures shown. Arrowheads indicate dead NTS. Arrows indicate LiS (early LiS in 5% and 10% HSe) and late liver stage (in 20% and 50% HSe). Results are pooled from three independent experiments with at least three biological replicates each. ×× p ≤ 0.01 comparing HM vs. 1% HSe; ┴┴ p ≤ 0.01 comparing HM vs. 5% HSe; ˅˅ p ≤ 0.01 comparing HM vs. 10% HSe; ≠≠ p ≤ 0.01 comparing HM vs. 20% HSe; ** comparing p ≤ 0.01 HM vs. 50% HSe. HSe, human serum; s.p., score points; p.t., post-transformation; SkS, skin stage; LuS, lung stage; LiS, liver stage.
(PZFX)

S5 File Efficient drug screening of advanced larval stages of *S*. *mansoni* generated in human serum. NTS were cultured in HM supplemented with 200 U/ml Penicillin and 200 μg/ml Streptomycin and 20% HSe for 1 week to generate LuS (B, D, F and H) and 6 weeks to obtain LiS (A, C, E, G) schistosomula. (A, B) Praziquantel (PZQ), (C, D) Oxamniquine (OXM), (E, F) Mefloquine (MFQ) and (G, H) Artemether (ART) were dissolved in DMSO and added at indicated concentrations for the entire duration of the experiment. 1% DMSO in culture medium served as control. Viability was scored before treatment (0 h) and 3 hours, 1, 2, 3 and 7 days a.t. Each data point is shown as a mean ± SD of three independent experiments with at least three biological replicates each. ××× p ≤ 0.001, ×× p ≤ 0.01, × p ≤ 0.05 comparing control with 1 μg/ml drug; ┴┴┴ p ≤ 0.001 ┴┴ p ≤ 0.01 ┴ p ≤ 0.05 control with 10 μg/ml drug; ***p ≤ 0.001, **p ≤ 0.01 *p ≤ 0.05 control with 100 μg/ml drug. LuS, lung stage; LiS, liver stage; s.p., score points; a.t., after treatment.
(PZFX)

S6 File Drug sensitivity is dependent on the developmental stage of *S*. *mansoni* larvae generated *in vitro*. NTS were cultured and matured in HM supplemented with 200 U/ml Penicillin and 200 μg/ml Streptomycin only or additionally supplemented with 20% HSe. 100, 10 and 1 μg/ml of (A) praziquantel, (B) oxamniquine, (C) mefloquine and (D) artemether were then added to the culture 1 day p.t. to test the SkS, 7 days p.t. to test the LuS and 6 weeks p.t. to test the LiS. Viability was scored 72h a.t. Each data point is shown as a mean ± SD of three independent experiments with at least three biological replicates each. ××× p ≤ 0.001, ×× p ≤ 0.01, × p ≤ 0.05 comparing control with 1 μg/ml drug; ┴┴┴ p ≤ 0.001, ┴┴ p ≤ 0.01, ┴ p ≤ 0.05 comparing control with 10 μg/ml drug; ***p ≤ 0.001, **p ≤ 0.01 *p ≤ 0.05 control with 100 μg/ml drug. SkS, skin stage; LuS, lung stage; LiS, liver stage; PZQ, praziquantel; OXM, oxamniquine; MFQ, mefloquine; ART, artemether; s.p., score points.
(PZFX)
