# Supplementary figures and images for: A novel cell-free method to culture Schistosoma mansoni from cercariae to juvenile worm stages for in vitro drug testing
Source: PLoS Negl Trop Dis. 2019 Jan 28;13(1):e0006590. doi: 10.1371/journal.pntd.0006590 (PMC6375649; doi:10.1371/journal.pntd.0006590)

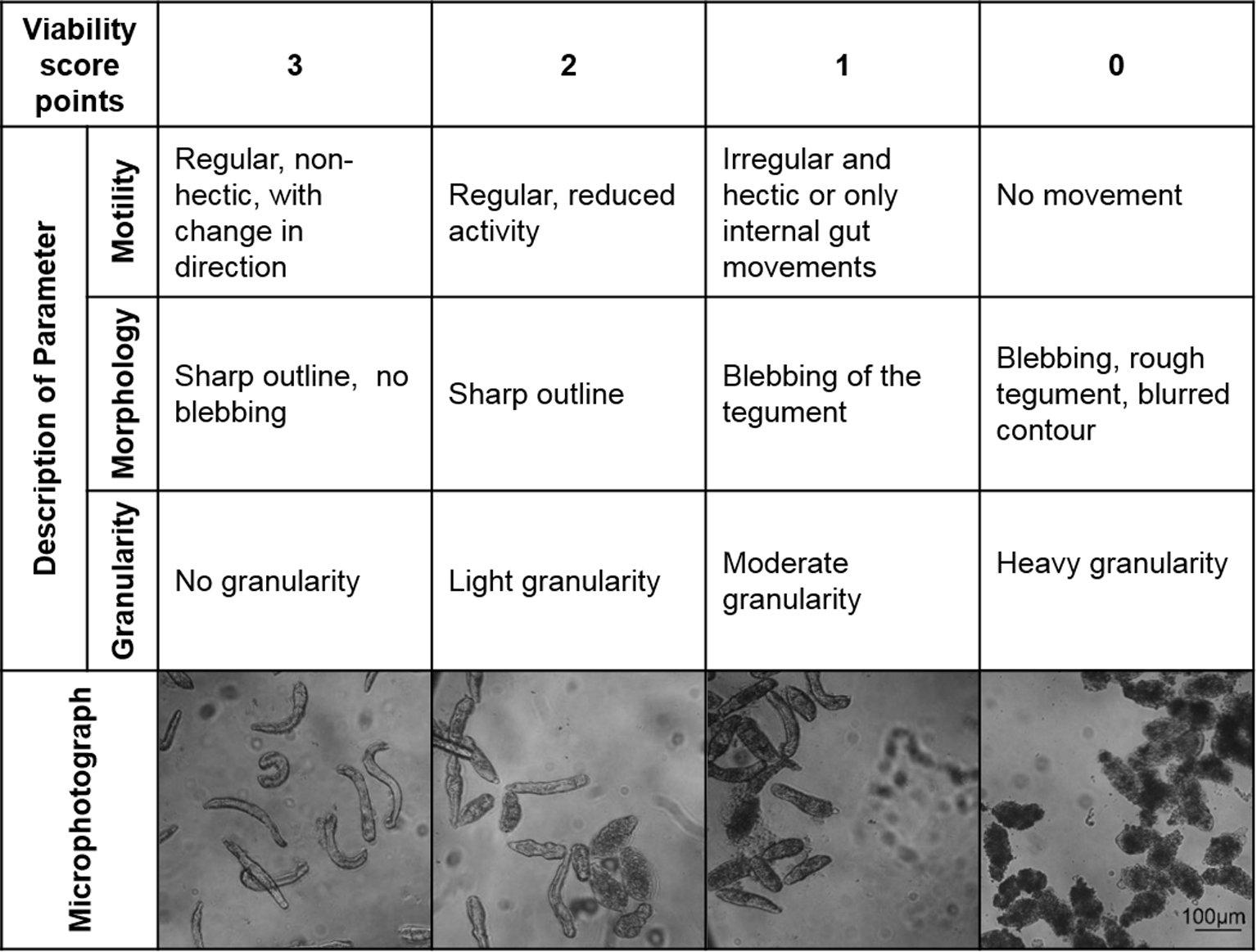

Supplement: S1 Fig — Photomicrographs represent indicated viability score points, which results from the overall assessment of motility, morphology and granularity of all NTS per well. Photomicrographs were taken on day 7 post-transformation using a digital camera fitted to an inverted microscope. Scale bar applies to all shown pictures. (TIF) [file pntd.0006590.s001.tif]

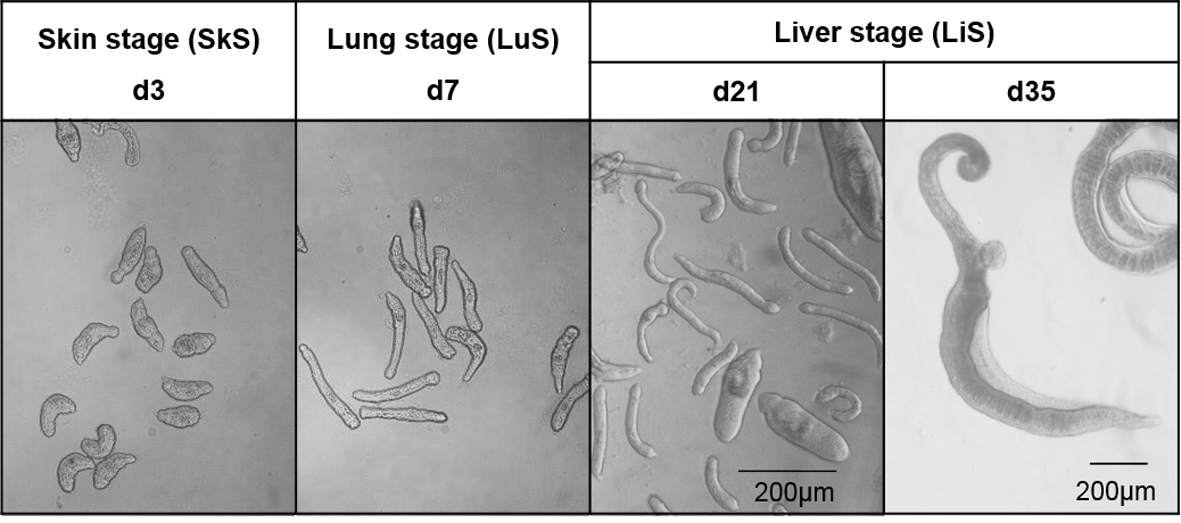

Supplement: S2 Fig — NTS were cultured in Hybrido Med (HM) supplemented with 200 U/ml Penicillin and 200 μg/ml Streptomycin and 20% human serum (HSe). Photomicrographs were taken at indicated time points. For skin, lung and day 21 liver stage the scale bar displayed in d21 LiS applies, whereas the scale bar for d35 only applies to this picture. (TIF) [file pntd.0006590.s002.tif]

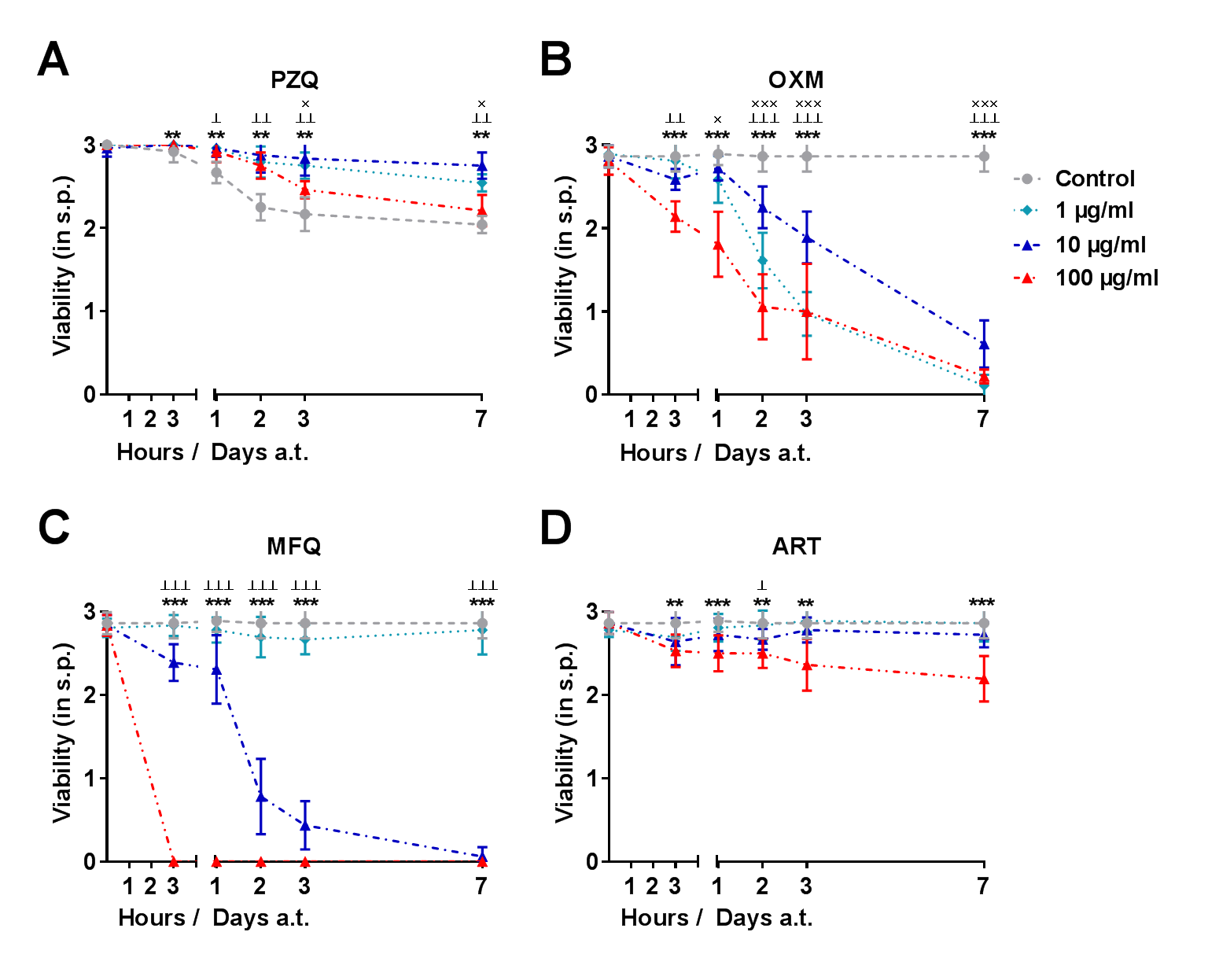

Supplement: S3 Fig — NTS were cultured in HM supplemented with 200 U/ml Penicillin and 200 μg/ml Streptomycin and 20% HSe. (A) PZQ, (B) OXM, (C) MFQ and (D) ART were dissolved in DMSO and added at 100, 10 and 1 μg/ml 24 h p.t. and the viability was scored at indicated time points. 1% DMSO in culture medium served as control. Each point is shown as a mean ± SD of three independent experiments with at least three biological replicates each ××× p≤0.001, ×× p≤0.01, × p≤0.05 ┴┴┴ comparing control with 1 μg/ml drug; p≤0.001 ┴┴ p≤0.01 ┴ p≤0.05 control with 10 μg/ml drug; ***p < 0.001, **p < 0.01 *p≤0.05 control with 100 μg/ml drug. s.p., score points; a.t., after treatment; PZQ, praziquantel; OXM, oxamniquine; MFQ, mefloquine; ART, artemether. (TIF) [file pntd.0006590.s003.tif]

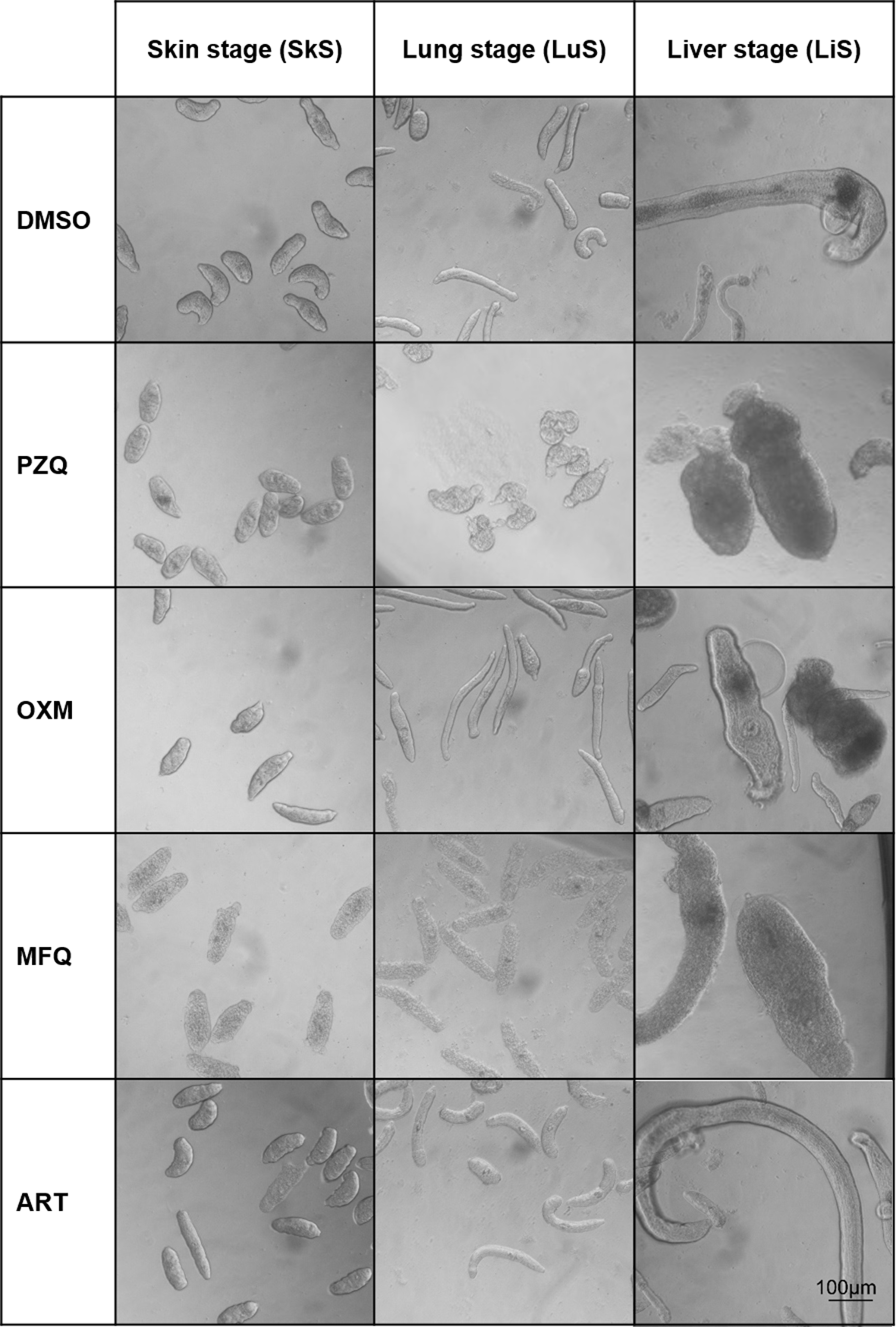

Supplement: S4 Fig — NTS were cultured in HM supplemented with 200 U/ml Penicillin and 200 μg/ml Streptomycin and 20% HSe. PZQ, OXA, MFQ and ART were dissolved in DMSO and added for a final concentration of 100 μg/ml to the culture, HM supplemented with 1% DMSO served as control. Photomicrographs were taken 24h after drug treatment. Scale bar applies to all shown pictures. PZQ, praziquantel; OXM, oxamniquine; MFQ, mefloquine; ART, artemether. (TIF) [file pntd.0006590.s004.tif]
